# Aplastic Anemia Associated with Oral Terbinafine: A Case Report and Review of the Literature

**DOI:** 10.4274/tjh.2013.0119

**Published:** 2014-12-05

**Authors:** Bülent Kantarcıoğlu, Hüseyin Kemal Türköz, Güven Yılmaz, Funda Pepedil Tanrıkulu, Işık Kaygusuz Atagündüz, Cafer Adıgüzel, Tülin Fıratlı Tuğlular

**Affiliations:** 1 Okmeydanı Training and Research Hospital, Clinic of Hematology, İstanbul, Turkey; 2 Marmara University Faculty of Medicine, Department of Pathology, İstanbul, Turkey; 3 Marmara University Faculty of Medicine, Department of Hematology, İstanbul, Turkey

**Keywords:** Onychomycosis, Terbinafine, Aplastic anemia, Hematological toxicity, Pancytopenia, Adverse events

## Abstract

Onychomycosis (OM) is a common fungal infection of the toenails and/or fingernails that is highly prevalent in the general population and also responsible for significant morbidity. OM is caused by dermatophytes, nondermatophytic molds, or yeast. Today systemic antifungal agents are considered as the gold standard for all types of OM. Here we report a case of aplastic anemia associated with oral terbinafine use and a review of the literature on hematological toxicities associated with terbinafine.

## Abstract (tr)

Onikomikoz (OM) el ve ayak tırnaklarının sık görülen fungal enfeksiyonudur. Genel toplumda prevalansı yüksek bir hastalık olması nedeniyle önemli morbiditeye yol açmaktadır. OM dermatofitler, nondermatofitik küf mantarları veya mayalar ile ortaya çıkan hastalıklardır. Günümüzde onikomikozun tedavisinde sistemik antifungal ajanlar tüm OM tiplerinde altın standart tedavi olarak kabul edilmektedir. Biz burada, oral terbinafin kullanımı sırasında gelişen bir aplastik anemi olgumuzu ve literatürde terbinafine ile ilişkilendirilmiş olan hematolojik toksisitelerin derlemesini sunuyoruz.

## INTRODUCTION

Onychomycosis is a very frequent fungal nail infection. The prevalence can be as high as 28%-40%, especially in elderly populations. Terbinafine is an antifungal agent with both fungicidal and fungistatic properties, which is highly effective and is the most frequently used agent in onychomycosis. Oral terbinafine is generally well tolerated with minimal reports of serious drug reactions. These rare adverse events are mostly reported as case presentations, and it is important to be familiar with them in order to be able to evaluate the risk and inform patients accordingly [1,2,3]. Here we report a case of aplastic anemia (AA) associated with oral terbinafine use and a review of the literature on hematological toxicities associated with terbinafine. Written informed consent was obtained from the patient and her husband for publication of this manuscript and accompanying images.

## CASE PRESENTATION

**Figure 1:**
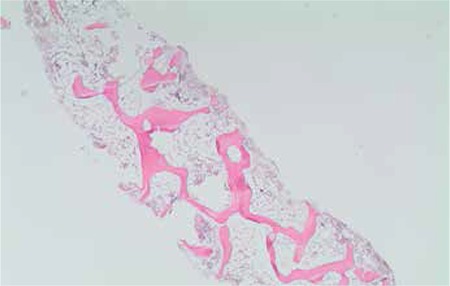
Bone marrow trephine biopsy: low cellularity in the bone marrow consistent with aplastic anemia (H&E, 20x).

**Figure 2:**
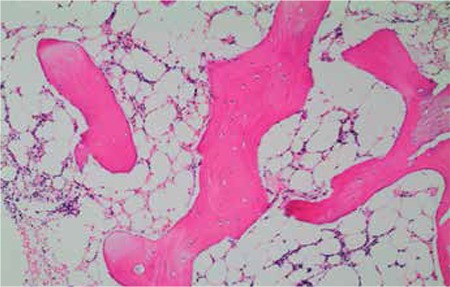
Bone marrow trephine biopsy: a few hematopoietic cells intermixed with lymphocytes and plasmocytes in interstitial areas (H&E, 100x).

A 41-year-old female presented with malaise, severe fatigue, nausea, and vaginal bleeding in April 2011. In her past history she was healthy, except that she reported taking terbinafine pills for 8 weeks for the treatment of longstanding recurrent toenail onychomycosis. She did not report any immune reactions or allergies to any drugs or substances. Her previous gynecological examination was normal, with a normal β-human chorionic gonadotropin level. Her complete blood count (CBC) revealed pancytopenia with white blood cell count of 3.2x109/L, absolute neutrophil count of 0.8x109/L, hemoglobin of 7.4 g/dL, and platelet count of 34x109/L. Her physical examination was unremarkable with no evidence of lymphadenopathy or organomegaly, except for a few petechiae and ecchymoses on bilateral legs. Peripheral blood smear was consistent with pancytopenia. Reticulocyte count was 0.7. Liver enzymes were elevated [AST: 61 U/L (N: 10-37 U/L), ALT: 117 U/L (N: 10-40 U/L), ALP: 434 U/L (N: 0-270 U/L), GGT: 471 U/L (N: 7-49 U/L)]. Renal function tests and lactate dehydrogenase were normal. Bone marrow aspiration and biopsy revealed severe reduction of all cell lineages without evidence of neoplastic infiltration, dysplasia, or fibrosis. The counted cellularity was 5% in bone marrow. Bone marrow karyotype analysis was normal. A gastroenterology consultation performed for the liver enzyme abnormalities did not provide an etiologic factor, pointing toward drug-induced hepatitis. Further work-ups, including levels of vitamin B12 and folate; neck, chest, and abdominopelvic computerized tomography; serology and polymerase chain reaction (PCR) tests for viral hepatitis, human immunodeficiency, Epstein-Barr virus, parvovirus B19, and cytomegalovirus; FLAER test for paroxysmal nocturnal hemoglobinuria; antinuclear antibody test; HLA-DRB1*15; and quantiferon test for tuberculosis, were all negative. The patient was diagnosed with AA, which was not severe at that time. Terbinafine treatment was stopped. Due to the use of a drug with probable hematologic toxicity, follow-up with supportive care was planned for the patient. During 3-4 weeks of follow-up time, blood values worsened with the need for erythrocyte and thrombocyte transfusions, in accordance with very severe AA (SAA). She did not have a matched related donor for transplantation. After confirming the diagnosis with a second bone marrow biopsy, she received rabbit antithymocyte globulin (ATG) plus cyclosporine-A (CYC). The clinical outcome after ATG + CYC was poor due to transient worsening of hematopoiesis and infectious complications. She spent 3 months in the hospital with perianal abscess, invasive aspergillosis, zoster virus reactivation, and several catheter infections. She required physical and psychological rehabilitation. Fortunately, the blood values began to recover at the end of the fourth month and full hematologic recovery was achieved at the end of the sixth month. The patient is still in complete remission after 18 months of ATG + CYC treatment (Figures 1 and 2).

## DISCUSSION AND REVIEW OF THE LITERATURE

Onychomycosis refers to all fungal infections of the nails. It is difficult to cure, has high recurrence rates, and can significantly affect a patient’s quality of life. Topical therapies are generally ineffective, and today treatment with systemic antifungal agents is accepted as the gold standard method for onychomycosis. In clinical trials, continuous terbinafine has repeatedly demonstrated higher efficacy when compared to other antifungal treatments. The recommended dosage for the treatment of onychomycosis is 250 mg/day orally for 12 weeks for toenails and 6 weeks for fingernails [1,2,3].

Oral terbinafine is generally well tolerated with minimal reports of serious drug reactions. Two large-scale postmarketing surveillance studies showed that the incidence of serious side effects was <1% [4,5]. In contrast, 2 studies of registry data from Austria and Denmark drew attention to blood dyscrasias associated with terbinafine [6,7]. Notable adverse events have been reported, including hepatitis requiring liver transplantation [8], drug-induced lupus reactions, severe skin reactions such as Stevens-Johnson syndrome, and, much less often, neutropenia/agranulocytosis and thrombocytopenia [9,10,11,12,13,14,15,16,17]. To our knowledge, ours is the first reported case of AA associated with terbinafine use.

AA is usually diagnosed within the setting of pancytopenia and hypocellular bone marrow when other diseases are excluded. SAA is almost always fatal if untreated. Once SAA is established, therapy should not be delayed in the hope of spontaneous recovery [18,19].

Many drugs have been associated with AA. The vast majority of patients exposed to these drugs do not develop AA and the reason for idiosyncratic reactions is unknown. AA can develop as a direct response to exposure, but it can also develop indirectly through immune-mediated mechanisms. P-glycoprotein (P-gp), the MDR-1 gene product, and the multidrug resistance-associated protein are energy-dependent transmembrane efflux pumps for a variety of lipophilic drugs. Underexpression of P-gp in normal cells might allow cytoplasmic accumulation of drugs and enhance their toxic effects. Two studies found that P-gp activity was decreased in patients with AA; levels were lowest in a subgroup with drug-induced AA [20,21]. In our case, the prolonged use of the highly lipophilic agent terbinafine might have caused direct toxicity, leading to AA.

Additionally, in a recently published study, the release of IL-8 and TNFα was significantly increased by treatment with terbinafine, which can explain how terbinafine may also cause immune-mediated injury [22]. Lupus-like reactions reported with terbinafine use may be an additional evidence of immune-mediated injury as an underlying mechanism. However, in most cases, the trigger of the mechanism of AA remains unclear. Historically, drug-induced AA has not been easily distinguished from idiopathic forms of the disease since causality is difficult to establish [23].

A review of hematologic toxicities associated with the use of terbinafine showed that the duration of terbinafine exposure leading to hematological toxicity is almost 1 month; the degree of cytopenia can be severe and patients mostly presented with infectious complications requiring hospitalization. While the clinical outcome was reversible in most cases, our patient required additional treatment with a high burden of risk and complications (Table 1).

In conclusion, keeping in mind the slow and persistent course of onychomycosis requiring long-term treatment, the high rate of success achieved with terbinafine, and the wide range of the population receiving terbinafine treatment, we advise detailed information of patients in regard to adverse events and we recommend monitorization of CBC at baseline and every month during terbinafine treatment. To our knowledge, this is the first case report of irreversible SAA following treatment with terbinafine that required immunosuppressive treatment with ATG + CYC. This case highlights the need for routine blood count monitoring during treatment with terbinafine. In these patients, clinicians should consider the rare incidence of SAA when there is agranulocytosis or pancytopenia.

**Conflict of Interest Statement**

The authors of this paper have no conflicts of interest, including specific financial interests, relationships, and/or affiliations relevant to the subject matter or materials included.

## Figures and Tables

**Table 1:**
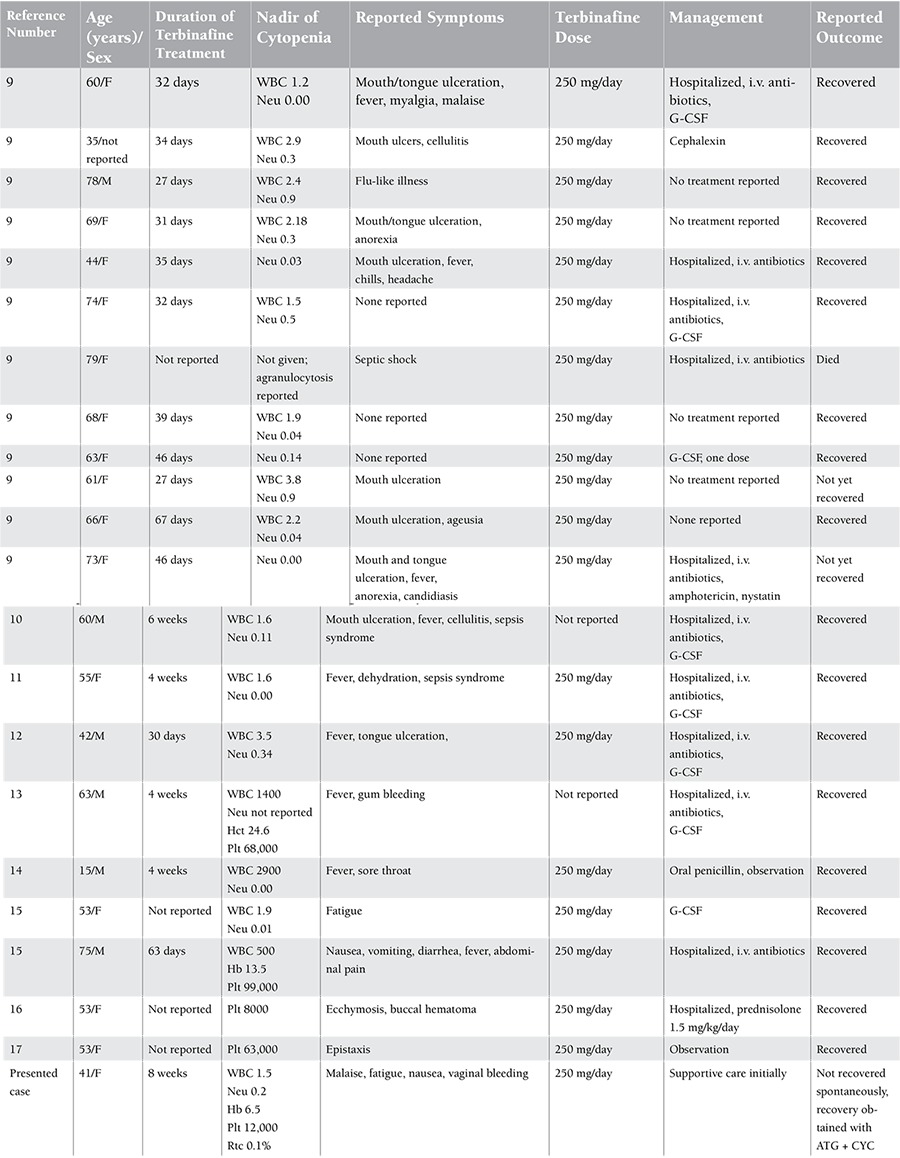
Reported cases of terbinafine-associated hematological toxicity in the literature.
